# Pedal-Assist Mountain Bikes: A Pilot Study Comparison of the Exercise Response, Perceptions, and Beliefs of Experienced Mountain Bikers

**DOI:** 10.2196/13643

**Published:** 2019-08-13

**Authors:** Cougar Hall, Taylor H Hoj, Clark Julian, Geoff Wright, Robert A Chaney, Benjamin Crookston, Joshua West

**Affiliations:** 1 Department of Public Health Brigham Young University Provo, UT United States; 2 Brigham Young University Provo, UT United States; 3 Department of Technology and Engineering Studies Brigham Young University Provo, UT United States

**Keywords:** public health, physical activity, heart rate

## Abstract

**Background:**

Mountain biking is an aerobic physical activity that has experienced rapid growth. The emergence of the electric pedal-assist mountain bike (eMTB), while not without its critics, presents the potential for an even larger segment of the population to enjoy the health benefits of mountain biking. Although the research focused on the use of e-bikes generally is growing, there is limited research specifically targeting eMTB use. Research is needed exploring the potential exercise response of riding an eMTB, together with the beliefs and perceptions of mountain bikers who have and have not experienced eMTB riding.

**Objective:**

This study aimed to compare conventional mountain bike and eMTB use. This was done by investigating 2 questions: (1) *What proportion of exercise response is retained for an experienced mountain biker while using an eMTB when compared with a conventional mountain bike?* and (2) *What are the perceptions and beliefs of experienced mountain bikers toward eMTBs both before and after riding an eMTB?*

**Methods:**

A convergent mixed methods data collection approach was used in the study. Participants completed both a pre- and postride questionnaire, and data regarding heart rate were collected. Heart rates from each ride were compared against each other.

**Results:**

The average heart rate during eMTB use was 94% (31/33) of the average heart rate during conventional mountain bike use. Therefore, eMTB use in this study achieved a majority of the exercise response and exceeded established biometric thresholds for cardiovascular fitness. Paired *t* test statistics were calculated to compare beliefs of conventional mountain bikes and eMTBs and to compare mean heart rate and speed between conventional mountain bike and eMTB use on the study loop. Participants overwhelmingly perceived the potential impact of eMTB use to be positive on both pre- and post-eMTB ride questionnaires.

**Conclusions:**

Despite the measured benefit, participants’ perceived exertion while riding the eMTB was low.

## Introduction

Promoting physical activity is an international public health priority [1,2]. The United States Department of Health and Human Services (HHS) recommends that adults engage in moderate aerobic physical activity for at least 150 min each week or vigorous aerobic physical activity for 75 min each week or a combination of both [3]. In spite of the recommendation, the Centers for Disease Control and Prevention (CDC) estimate that only 20.9% of adults in the United States fulfill the recommendation [4]. There are many reasons attributed to the disregard, and potential solutions have been implemented and studied. This study investigated the physical activity of electric pedal-assist mountain biking as a viable solution to improve compliance with HHS recommendations.

Mountain biking is an aerobic physical activity that has experienced rapid growth in the United States [5]. However, mountain biking is often limited or perceived to be limited to those individuals who already enjoy a relatively high level of cardiovascular fitness and endurance. The emergence of electric pedal-assist bikes (e-bikes), and specifically electric pedal-assist mountain bikes (eMTB), presents an opportunity for a larger segment of the population to enjoy the health benefits of mountain biking [6]. A review of e-bike literature supports the hypothesis that e-bike use is a beneficial physical activity for a wide range of individuals with an added benefit of promoting health among individuals otherwise reluctant to engage in physical activity [7-12]. Recent studies suggest that e-bike commuting may be helpful in improving glucose tolerance [10], decreasing perceived exertion and improved enjoyment [11], and reducing barriers to conventional cycling, including commuting [13]. For example, results from a Web-based survey demonstrated that those using an e-bike to ride to work report an ability to ride greater distances while perspiring less, suggesting that e-bikes may reduce some of the personal barriers of conventional cycling as a form of active transport [14]. This combined body of research shows the potential physical health benefits of e-bikes.

A typical e-bike has an electric motor that functions as a pedal-assist, only engaging when the individual pedals. The motor's contribution allows a rider to cycle greater distances and up steeper terrain because of the decreased physical workload [14]. Though heart rate, energy expenditure, oxygen consumption, and intensity is generally lower compared with a conventional bike [7,13], e-bike use still produces moderate physical activity in comparable settings and between groups with differing fitness levels [8,9].

Although the research focused on the use of e-bikes is growing, there is limited research regarding eMTB use. There are 2 studies that investigated heart rate and energy expenditure between e-bike use with conventional bikes [7,13]. Each found that energy use was likely lower with e-bikes. Nevertheless, findings indicate that an e-bike rider still pedals and exerts energy, which may help them meet the physical activity guidelines and gain the associated health benefits. Part of our inquiry is to test this observation with eMTBs, which has not been done previously.

Although the popularity of e-bikes is growing and their benefits related to active transport and physical activity for a broad segment of the population are being established, the introduction of eMTBs to the mountain biking community has been met with much resistance. Concerns have been raised concerning eMTB use and increased trail damage, increased conflict between trail users, a potential for decreased trail access, and the perception that pedal-assist mountain bikes are akin to motorcycles and do not represent *real* mountain biking. These concerns have the potential to limit the adoption of eMTBs by individuals who may benefit from them or otherwise enjoy their use. To date, researchers are yet to explore any aspect of eMTB use, including the potential exercise response of riding an eMTB, as well as the beliefs and perceptions of mountain bikers who have and have not experienced eMTB riding. The purpose of this pilot study was to compare conventional mountain bike and eMTB use. In particular, this study aimed to address 2 research questions: (1) *What proportion of exercise response is retained for an experienced mountain biker while using an eMTB when compared with a conventional mountain bike?* and (2) *What are the perceptions and beliefs of experienced mountain bikers toward eMTBs both before and after riding an eMTB?*

## Methods

### Participants

Experienced mountain bikers aged between 18 and 65 years were recruited to participate in this study. Exclusion criteria included non–mountain bikers and mountain bikers with the inability to engage in moderate to vigorous intensity mountain biking for 12 miles or those who have a medical condition that would prevent them from moderate to vigorous exercise.

### Procedures

The institutional review board at Brigham Young University approved this study. A study announcement was posted to a regional Facebook page popular with local mountain bikers. Individuals wishing to participate were directed to contact the principal investigator via email and set up a time to meet at a local trail system. Upon arrival at the trail system, individuals completed the pre-eMTB ride questionnaire using Qualtrics, a Web-based survey software platform, on their personal phone or the principal investigator’s laptop computer. The first pre-eMTB questionnaire item included obtaining the individual’s informed consent to participate in the study. Consenting individuals then proceeded to the remainder of the questionnaire. Upon completing the pre-eMTB ride questionnaire, participants were fitted with a heart rate monitor and corresponding Apple Watch. Each Apple Watch was paired to the heart rate monitor and Strava app to record the participant’s ride data, including global positioning system (GPS) tracking, total distance traveled, and speed traveled. Next, participants were randomly assigned to ride the 6-mile study loop on either a conventional mountain bike or an eMTB. The study loop included approximately 700 feet of elevation gain spread throughout the ride with the most intense climbing section averaging a 5% incline over a distance of 1 mile. Upon completing the study loop on their initially assigned bike, participants’ heart rate and Strava data were saved. Participants then rode the loop again on the remaining bike—whichever type of bike they did not ride while completing the first loop. After completing the study loop a second time, participant heart rate and Strava data were again saved and each participant then completed the post-eMTB ride questionnaire. The study was completed between May 24 and June 16, 2018.

### Instruments/Measurements

Both conventional mountain bikes and eMTBs were used in this study to establish a comparison between participants’ heart rate and speed while riding the study loop. The electric mountain bikes used were Class 1 pedal-assist 2017 Specialized Turbo Levo FSR Comp Carbon 6Fattie models with a maximum assistance speed of 20 mph (32 kph) [15]. Participants were given the option of either riding their own traditional mountain bike or a 2017 Specialized Stumpjumper FSR Comp 6Fattie model—the equivalent of the Turbo Levo model without pedal-assist—while completing the non-eMTB lap of the study loop.

Third-generation Apple brand watches (Apple Watch) were paired with Polar H10 heart rate monitors to record the participants’ continuous heart-rate data while completing each lap of the study loop. Total distance, speed, and time while riding was recorded during study laps using Strava, a mobile app using GPS technology available via the App Store for iOS and Apple Watch platforms. A comparison of participants’ heart rate was used as a proxy measure to estimate exercise response. Specifically, estimated maximum heart rate (MHR) was calculated by subtracting the age of the study participants from 220. The estimated MHR was then used to establish a target average heart rate range for moderate-intensity physical activity (50%-70% of MHR) and vigorous-intensity physical activity (70%-85% of MHR). These ranges were calculated based on target heart rate recommendations from the CDC and the American Heart Association [16,17].

A total of 2 survey instruments, developed using the Web-based survey software provided by Qualtrics, were used in this study. Survey 1—the pre-eMTB ride questionnaire administered before riding either of the study bikes or loops—was used to gather basic demographic information, mountain biking history and experience data, perceived impact of eMTB use, and beliefs about eMTBs. Survey 2—the post-eMTB ride questionnaire—was administered after participants had completed riding the study loop on both a conventional mountain bike and an eMTB. The questions in Survey 2 were identical to the final questions asked in Survey 1, targeting participants’ perceptions and beliefs related to eMTB use.

### Analysis

All statistical analyses were performed using SAS version 9.4 (SAS Institute Inc). Descriptive statistics were used to summarize demographic data from Survey 1. Paired *t* test statistics were calculated to compare beliefs about conventional mountain bikes and eMTBs and to compare mean heart rate and speed between conventional mountain bike and eMTB use on the study loop.

## Results

### Demographics

The majority of participants were male (88%; 29/33), and all identified as non-Hispanic and white. The average age was just under 38 years. All participants had completed at least some college. Complete demographic and mountain biking experience information can be found in Tables 1 and 2. Approximately half (16/33) of participants had more than 10 years of mountain biking experience. The majority (24/33) reported mountain biking at least twice each week. All participants indicated they mountain bike to increase fitness, spend time outdoors, and recreate or have fun. Few participants (n=3) had previously ridden an eMTB before participating in this study.

### Exercise Response

Table 3 provides a comparison of average distance, time, speed, and heart rate metrics between conventional mountain bike and eMTB use as well as paired *t* test results.

Participants traveled approximately 5.5 miles (8.85 km) while riding the study loop. A paired *t* test analysis (Table 3) revealed participants completed the course an average of 12 min and 40 seconds faster when riding the eMTB as opposed to the conventional mountain bike (*P*<.001). The average speed of travel on the eMTB was 4.1 mph (6.6 km/h) faster than on the conventional mountain bike (*P*<.001). Participants’ average heart rate during the eMTB ride was 9.9 beats per minute (bpm) lower than during the conventional mountain bike ride (*P*<.001). With a mean age of 37.8 years, participants’ estimated MHR was 182 bpm. The target heart rate zone for moderate-intensity exercise (50%-70% of MHR) and vigorous-intensity exercise (70%-85% of MHR) was then calculated to be 91 bpm to 127 bpm (0.5x182=91.12, 0.7x182=127.4) and 128 bpm to 155 bpm (0.7x182=127.4, 0.85x182=154.7), respectively [16]. Riding both the conventional mountain bike and the eMTB placed participants’ in the upper half of the vigorous-intensity zone (Table 4).

**Table 1:** Demographics (N=33).

Demographics			Value, n (%)
**Age (years)**			
	20-29	7 (21)	
	30-39	9 (27)	
	40-49	13 (39)	
	50 and older	4 (12)	
**Race**			
	White	33 (100)	
**Ethnicity**			
	Non-Hispanic or Latino	33 (100)	
**Sex**			
	Male	29 (88)	
	Female	4 (12)	
**Education level**			
	Some college (not graduated)	8 (24)	
	2-year college degree	6 (18)	
	4-year college degree	12 (36)	
	Master’s degree	5 (15)	
	Doctoral degree	2 (6)	
**Annual household income ($ US)**			
	Less than 30,000	3 (9)	
	40,000-49,999	2 (6)	
	50,000-59,999	3 (9)	
	60,000-69,999	2 (6)	
	70,000-79,999	3 (9)	
	80,000-89,999	3 (9)	
	90,000-99,999	1 (3)	
	100,000 or more	16 (48)	

**Table 2:** Mountain biking experience (N=33).

Mountain biking experience		Value, n (%)
**Mountain biking experience^a^ (years)**		
	Less than 1	2 (6)
	1-5	7 (23)
	6-10	6 (19)
	11 and more	16 (52)
**During a typical riding season, how often do you mountain bike?**		
	1-2 days a month	3 (9)
	Once a week	6 (18)
	2-3 days a week	19 (58)
	4-5 days a week	5 (15)
	Daily	0 (0)
**For which of the following reasons do you ride a mountain bike? (yes)**		
	Recreation or fun	33 (100)
	To spend time with family	16 (48)
	To increase fitness	33 (100)
	Racing	3 (9)
	To spend time with friends	29 (88)
	To spend time outdoors	33 (100)
**What best describes your bike?**		
	Cross-country	5 (15)
	Trail	11 (33)
	All mountain/Enduro	17 (52)
Has previously ridden a class 1 electric pedal-assist mountain bike		3 (9)

^a^N=31.

**Table 3:** Riding and exercise response results.

Comparison of distance, time, speed, and heart rate metrics (N=33)	Descriptive statistics		Paired *t* test: MTB^a^ vs eMTB^b^	
	MTB, mean (SD)	eMTB, mean (SD)	Mean difference	*P* value
Time (min:seconds)	38:54 (7:48)	26:14 (3:45)	12:40	<.001
Average speed (miles per hour)	8.8 (1.4)	12.9 (1.7)	−4.1	<.001
Average heart rate (beats per minute)	154.8 (12.9)	144.9 (13.7)	9.9	<.001

^a^MTB: mountain bike

^b^eMTB: electric pedal-assist mountain bike

**Table 4:** Riding and exercise response results.

Comparison of distance, time, speed, and heart rate metrics (N=33)	MTB^a^, n (%)	eMTB^b^, n (%)	*P* value^c^
Moderate-intensity physical activity	2 (6.1)	4 (12.1)	.09
Vigorous-intensity physical activity	31 (93.9)	29 (87.9)	—^d^

^a^MTB: mountain bike

^b^eMTB: electric pedal-assist mountain bike

^c^Chi-Square: MTB vs eMTB.

^d^Not applicable.

### Perceptions

Table 5 includes pre- and post-eMTB ride data related to perceptions of potential impacts of eMTB use. Participants overwhelmingly perceived the potential impact of eMTB use to be positive on both pre- and post-eMTB ride questionnaires. Only “Potentially allows riders to ascend or climb greater distances and elevations in less time on dirt trails” was significantly different on the post-eMTB ride questionnaire, with more participants in agreement that eMTB use would have such an impact.

### Beliefs

Table 6 includes the results of 26 pre- and post-eMTB ride belief statements regarding eMTB use. A total of 4 belief statements were significantly different after riding the eMTB. Fewer participants agreed that *eMTB use will prove to be a passing fad* and that they *could get the same cardiovascular workout on an eMTB as a conventional mountain bike*, whereas more participants agreed that their *heart rate is considerably lower while riding an eMTB as compared with a conventional mountain bike* and *eMTB use allows riders greater and deeper access to backcountry dirt trails.* Table 7 includes results from the final questionnaire item asking *how beliefs and perceptions about eMTBs changed after riding one* showed that few participants (n=3) were less accepting of eMTBs, some experienced no change (n=8), and the majority (n=20) were more accepting of eMTBs after riding one.

**Table 5:** Perceptions of potential impact of electric pedal-assist mountain bike use (N=32).

Perceptions of potential impacts of electric pedal-assist mountain bike use	Preride (agreed), n (%)	Postride (agreed), n (%)	*P* value^a^
Potentially allows older riders to continue enjoying mountain biking on dirt trails	32 (100)	30 (94)	.16
Potentially allows less-fit riders to more fully enjoy mountain biking on dirt trails	27 (84)	27 (84)	>.99
Potentially allows injured or disabled riders to continue enjoying mountain biking on dirt trails	32 (100)	31 (97)	.33
Potentially allows riders of varying fitness levels to mountain bike together on dirt trails	25 (78)	26 (81)	.66
Potentially allows all riders to mountain bike longer distances on trails^b^	25 (81)	27 (87)	.33
Potentially allows riders greater and deeper access to the backcountry on dirt trails	25 (78)	28 (88)	.18
Potentially allows riders to ascend or climb greater distances and elevations in less time on dirt trails	23 (72)	29 (91)	*.03*
Potentially allows riders who may otherwise shuttle the ascent or drive to the top of the trail in a vehicle to ride up on dirt trails	27 (84)	27 (84)	>.99
Potentially increases the appeal of riding on dirt trails to more people	21 (66)	24 (75)	.33
Potentially improves public health outcomes by increasing rates of physical activity	27 (84)	27 (84)	>.99

^a^*P* values were derived from paired *t* tests of preride and postride values. Variables were coded using the following logic: 0=Negative (con), 1=Positive (pro). The significant *P* value (<.05) has been italicized.

^b^N=31.

**Table 6:** Beliefs regarding electric pedal-assist mountain bike use (N=33).

Beliefs regarding eMTB^a^ use	Preride (agreed), n (%)^b^	Postride (agreed), n (%)^b^	*P* value ^c^
I believe riding an eMTB is cheating	16 (48)	13 (39)	.11
I believe riding an eMTB is equivalent to riding a motorcycle	4 (12)	5 (15)	.38
I believe if eMTBs are allowed on existing dirt trails, then trail access for all mountain bikers will be compromised	15 (45)	10 (30)	.26
I believe eMTB riders perceive they are actually mountain biking, but they are not; eMTB use is not mountain biking	11 (33)	5 (15)	.23
I believe eMTBs should be banned from existing mountain bike trails and trail systems	6 (18)	6 (18)	.79
I believe eMTB use causes more trail damage compared with conventional mountain bikes	6 (18)	4 (12)	.70
I believe eMTB use should be limited to riders with physical handicaps or impairments	6 (18)	5 (15)	.08
I believe that in the future, eMTB use will replace conventional mountain biking^d^	2 (6)	4 (13)	.26
I believe eMTBs have the potential to help older riders continue to enjoy mountain biking	32 (97)	31 (94)	.60
I believe eMTBs have the potential to help less-fit riders increase their fitness levels and transition to conventional mountain biking	25 (76)	25 (76)	.71
I believe I could get the same cardiovascular workout on an eMTB as I do my conventional mountain bike	14 (42)	5 (15)	*.002*
I believe my heart rate is considerably lower while riding an eMTB as compared with my conventional mountain bike	18 (55)	28 (85)	*<.001*
I am opposed to eMTB use	6 (18)	4 (12)	.11
I believe eMTBs are primarily being pushed on cyclist by the industry to make money	5 (15)	7 (21)	.41
I believe eMTB use will have a negative impact on mountain biking	7 (21)	7 (21)	.25
I believe eMTB use will prove to be a passing fad	10 (30)	6 (18)	*.03*
I am opposed to eMTB use by healthy individuals	8 (24)	8 (24)	.45
I am opposed to eMTB use on the same trails as conventional mountain biking	7 (21)	7 (21)	.32
I am fine with pedal-assist bike use on the street, but I am opposed to their use on dirt trails	7 (21)	6 (18)	.14
I believe eMTB use allows riders of varying fitness levels to mountain bike together on dirt trails	30 (91)	26 (79)	.34
I believe eMTB use allows all riders to bike longer distances	32 (97)	33 (100)	.07
I believe that eMTB use allows riders greater and deeper access to backcountry dirt trails	30 (91)	32 (97)	*.03*
I believe that eMTB use allows riders to ascend or climb greater distances and elevations in less time on dirt trails	31 (94)	33 (100)	*.001*
I believe that eMTB use allows riders who may otherwise shuttle the ascent or drive to the top of the trail in a vehicle to ride up on dirt trails	31 (94)	31 (94)	.54
I am supportive of eMTB use^e^	26 (84)	26 (84)	.17

^a^eMTB: electric pedal-assist mountain bike.

^b^Agreed n (%) includes both *strongly agree* and *agree* responses.

^c^*P* values were derived from paired *t* tests of preride and postride values. Variables were coded using the following logic: 1=strongly disagree, 2=disagree, 3=agree, and 4=strongly agree. Significant *P* values (<.05) are italicized.

^d^N=32.

^e^N=31.

**Table 7:** Overall belief and perception surrounding the question: how have your beliefs and perceptions about eMTBs^a^ changed after riding one? (N=33).

Overall belief and perception	Value, n (%)
I am less accepting of eMTBs after riding one	3 (9)
My beliefs and perceptions have not changed at all	8 (24)
I am more accepting of eMTBs after riding one	20 (61)
Other	2 (6)

^a^eMTB: electric pedal-assist mountain bike.

## Discussion

### Principal Findings

This study sought to address 2 research questions: (1) *What proportion of exercise response is retained for an experienced mountain biker when using an eMTB compared with a conventional mountain bike?* and (2) *What are the perceptions and beliefs of experienced mountain bikers toward eMTB both before and after riding an eMTB?* Although significant differences in heart rate were measured between conventional mountain bike use and eMTB use, riding the study loop on both types of mountain bikes placed the vast majority of participants in the vigorous-intensity heart rate zone. Using heart rate as a proxy measure for cardiovascular exercise intensity and related exercise response, eMTB use appears to be an excellent form of aerobic or cardiovascular exercise, even for experienced mountain bikers who regularly engage in this fitness activity. *Physical Activity Guidelines for Americans* established by the CDC indicate that for substantial health benefits, adults should engage in at least 150 min a week of moderate-intensity aerobic physical activity or 75 min a week of vigorous-intensity aerobic physical activity [3,16]. Average heart rate during eMTB use was 93.6% of average heart rate during conventional mountain bike use. Riding both types of bikes on the study loop caused the participants to exceed at least heart-rate levels for moderate-intensity fitness activities and placed the average heart rate for a majority of participants in the vigorous-intensity zone [16]. Therefore, eMTB use in this study retained the bulk of the exercise response and exceeded established biometric thresholds for cardiovascular fitness. These findings of eMTB use on soft-surface trails are comparable to recent findings using e-bikes on city bike paths in which it was estimated that 95.5% of the cardiovascular benefit of conventional bike use was retained [18]. Although findings from the extant literature indicate that e-bikes can generally satisfy requirements for moderate-intensity physical activity [7-11,13,19], this study is the first to explore the exercise response of eMTB use on soft-surface trails and the first to associate pedal-assist bikes with vigorous exercise.

Although eMTB use provided an intense cardiovascular workout in this study, average riding speed on the eMTB was approximately 4 mph (approximately 6.5 kph) faster than speeds on the conventional mountain bike, resulting in less time needed to complete the study loop. If a conventional mountain bike was to be replaced by an eMTB as part of a cardiovascular fitness program, then total ride time, not ride distance, would need to remain constant. In this study, speed was presented as an average across the entire study loop. It is possible that the higher speed for eMTBs is a factor in forming attitudes and beliefs both for and against their use. For example, higher eMTB speeds in high traffic areas or up hills may be a perceived source of trail conflict and slower eMTB speeds on downhill trail sections may result in trail congestion. These examples are only speculative and could be tested in future research on the adoption and uptake of eMTBs.

This study represents the first attempt to measure perceptions and beliefs of experienced mountain bikers before and after riding an eMTB. Relatively few significant attitudinal changes occurred from preride to postride, likely because of a sample of participants holding positive attitudes about eMTB at the onset. Only 18% of participants indicated they were opposed to eMTB on the preride survey. As there are many in the mountain biking community with strong negative opinions about eMTBs [6], this is likely a reflection of sampling bias, which is to say that those volunteering to participate in this study likely had more positive views of eMTBs and were excited for the opportunity to ride one. There were, however, several significant findings related to attitudes and beliefs along with several nonsignificant findings worthy of discussion.

After riding an eMTB, attitudes related to the future of eMTB use changed with fewer participants considering eMTBs to be a passing fad. This shift is consistent with industry trends and forecasts as eMTB sales climbed to US $77.1 million in 2017, a 91% increase in US sales from the previous year and an 8-fold increase since 2014 [20,21]. Market predictions are that eMTB sales will represent approximately 30% of the mountain biking market by 2020 [22].

Of particular note, participants in this study did not perceive riding an eMTB to be a workout or taxing on their cardiovascular system. Although mean heart-rate data indicated the eMTB study loop resulted in an approximate 10 bpm reduction when compared with the conventional mountain bike, all participants reached at least moderate levels of intensity and most reached vigorous levels while riding the eMTB. Despite this, participants’ perceived exertion while riding the eMTB was low. This finding has potential implications for the utility of eMTBs in helping all users, including the experienced mountain bikers in this study as well as more sedentary individuals, to engage in regular physical activity and meet physical activity guidelines. As key constructs of the Health Belief Model (HBM), both *perceived benefits* and *perceived barriers* are predictive of adherence to health recommendations and behavior change [23]. *Perceived benefits* specifically refer to one’s opinion of the efficacy of an advised action to reduce health risks [23]. *Perceived barriers* refer to one’s opinion of the cost, whether psychological, physiological, or financial, of engaging in a health-promoting behavior or practice [23]. The low perceived exertion of riding an eMTB, together with the cardiovascular benefit of continuous target heart rate zone activity, make the total perceived benefits of eMTB riding high and the perceived barriers low. This has been observed as it relates to physical activity in general, where perceptions of exertion significantly impact activity levels [24,25]. Utilizing pedal-assist technology to decrease the perceived exertion of physical activity may be a critical catalyst in helping individuals become more physically active. Specifically pertaining to the uptake of e-bikes, lower perceived exertion has been reported as impactful [26]. In relation to the HBM, this study examined the physiological barriers and benefits of eMTB use, but other barriers may exist that could delay the uptake of this technology. It is possible that on account of being an emerging technology and with the addition of an electric motor, potential users of eMTBs perceive the financial cost of purchasing an eMTB too high. Indeed, high performance eMTBs can be costly. The extent to which these perceptions exist and how they might impact potential riders was beyond the scope of this study but could be studied in the future.

Participants were more accepting of eMTBs after riding one. The adage “don’t knock it until you try it” appears applicable with pedal-assist technology. A recent qualitative analysis of eMTB threads in mountain biking forums concluded that individuals could be divided into 2 groups when commenting on eMTBs: those who had personal experience with an eMTB and those who did not. The authors concluded that inexperience with an eMTB appears central to the conflict surrounding eMTB use and that many misconceptions about what an eMTB *is* and *can do* are resolved by riding one [6]. This study found that most participants either became more accepting (61%) of eMTBs after riding one or reported no change (24%) in their level of acceptance.

Of interest in this study are the perceptions and beliefs that were not significantly altered by the experience of riding an eMTB. Overwhelming agreement existed at both pre- and postride data collection related to eMTBs’ ability to help older and less-fit riders find enjoyment in riding. Another stable perception is that eMTBs have the potential to improve public health outcomes through the encouragement or promotion of physical activity. Future research should explore this potential by including sedentary, less active, overweight or obese, and older individuals as participants. Such investigations could target behaviors, attitudes, and biometric indicators longitudinally.

### Limitations

Findings from this study should be interpreted with consideration of several limitations. This study was limited by its small sample. Although the sample size in this study is equal to or greater than similar studies of pedal-assist bikes, it is not sufficiently large to generalize or draw conclusions beyond this specific sample. In addition, this study used heart-rate data as a proxy measure for exercise response and cardiovascular exercise intensity. Future studies examining similar variables would benefit from more sophisticated measures, such as maximal oxygen uptake, metabolic equivalents, and watts. Likewise, participants had only 1 trial on the eMTB and their heart-rate response might have changed after an extended observation period. Finally, the sampling procedure employed to recruit experienced mountain bikers in this study yielded participants who might have already been largely supportive of eMTB use. A more random sample may have produced different results, especially related to perceptions and beliefs before and after riding an eMTB.

### Conclusions

This is the first study to compare the exercise response of conventional mountain bike and eMTB use on soft-surface trails and the first to associate pedal-assist bikes with vigorous-intensity aerobic or cardiovascular fitness. Findings indicate that riding an eMTB is moderate to vigorous physical activity, providing individuals with the opportunity to meet physical activity guidelines. Findings related to perceptions and beliefs before and after riding eMTBs were mixed yet support the use of pedal-assist technology in promoting physical activity.

## Abbreviations

bpm: beats per minute
CDC: Centers for Disease Control and Prevention
e-bike: electric pedal-assist bike
eMTB: electric pedal-assist mountain bike
GPS: global positioning system
HBM: Health Belief Model
HHS: Health and Human Services
MHR: maximum heart rate
